# Hypoxemia is an independent predictor of bronchiolitis obliterans following respiratory adenoviral infection in children

**DOI:** 10.1186/s40064-016-3237-7

**Published:** 2016-09-20

**Authors:** Pei-Qiong Wu, Xing Li, Wen-Hui Jiang, Gen-Quan Yin, Ai-Hua Lei, Qiang Xiao, Jian-Jun Huang, Zhi-Wei Xie, Li Deng

**Affiliations:** 1 Department of Respiration, Guangzhou Women and Children’s Medical Center, Guangzhou Medical University, 9 Jinsui Road, Guangzhou, 510623 People’s Republic of China; 2 Institute of Human Virology, Zhongshan School of Medicine, Sun Yat-Sen University, 74 Zhongshan 2nd Road, Guangzhou, 510080 People’s Republic of China; 3 Department of Medical Oncology, The Third Affiliated Hospital of Sun Yat-sen University, 600 Tianhe Road, Guangzhou, 510630 People’s Republic of China

**Keywords:** Hypoxemia, Bronchiolitis obliterans, Adenovirus, Respiratory infection

## Abstract

Bronchiolitis obliterans (BO) is an uncommon and severe sequela of chronic obstructive lung disease in children that results from an insult to the lower respiratory tract. Few prognostic factors achieved worldwide acknowledgment. In the present study, we retrospectively collected the children with respiratory adenoviral infection and identified the predictive factors of BO. In the period between Jan 2011 and December 2014, the consecutive in-hospital acute respiratory infection children with positive result for adenovirus were enrolled into the present study. High resolution computerized tomography and clinical symptoms were utilized as the diagnostic technique for BO. Multivariate analysis using a Logistic proportional hazards model was used to test for independent predictors of BO. A total of 544 children were included with 14 (2.57 %) patients developed BO. Compared with children without BO, BO children presented higher LDH (523.5 vs. 348 IU/ml, *p* = 0.033), lower blood lymphocyte count (2.23 × 10^9^/L vs. 3.24 × 10^9^/L, *p* = 0.025) and higher incidence of hypoxemia (78.6 vs. 20.8 %, *p* = 0.000). They presented relatively persistent fever (15.5 vs. 7 days, *p* = 0.000) and needed longer treatment in hospital (19.5 vs. 7 days, *p* = 0.000). Concerning treatment, they were given more intravenous γ-globulin (85.7 vs. 36.8 %, *p* = 0.000), glucocorticoids (78.6 vs. 24.3 %, *p* = 0.000) and mechanical ventilation (35.7 vs. 5.5 %, *p* = 0.001). Multiple analyses determined that hypoxemia was the only independent predictor for BO. The present study identified hypoxemia as the independent predictive factor of BO in adenoviral infected children, which was a novel and sensitive predictor for BO.

## Background

Bronchiolitis obliterans (BO) is an uncommon and severe sequela of chronic obstructive lung disease in children that results from certain insult to the lower respiratory tract. It is featured by tachypnoea, increased anteroposterior chest diameter, crackles, wheezing, and hypoxaemia for at least 30 days after the initial insult. Luminal obstruction with inflammation, granulation tissue, fibrosis, obliteration of the small airways and bronchiectasis are the pathological characteristics (Li et al. 2014; Champs et al. 2011; Xie et al. 2014; Bosa et al. 2008; Mosquera et al. 2014). Due to its poor prognosis and shortage of effective remedy, identification of predictive factors for BO became one of hottest topic in this field. However, few prognostic factors achieved worldwide acknowledgment (Murtagh et al. 2009; Colom and Teper 2009; Yalcin et al. 2003; Khalifah et al. 2004). The latent causes might be the heterogenicity of this disease.

BO was a heterogeneous syndrome due to multiple causes. The reported causes were indicated as acute rejection (El-Gamel et al. 1999), lymphocytic bronchitis (Husain et al. 1999), cytomegalovirus pneumonitis (Heng et al. 1998), adenoviral infection (Murtagh et al. 2009), single lung transplant (Hadjiliadis et al. 2002), anti-human leukocyte antigen antibody development (Palmer et al. 2002), and et al. (Champs et al. 2011). The predictors for BO in various backgrounds shall be divergent widely due to different latent mechanisms. Thus prognostic analysis of BO shall be based on a single cause. Adenoviral infection was identified as the major cause for BO in children (Champs et al. 2011; Murtagh et al. 2009; Khalifah et al. 2004). Thus, it is imperative to investigate the predictors for BO following respiratory adenoviral infection in children.

In our Women and Children’s Medical Center, a 1400 bed tertiary children’s hospital located in Southern China with a service population of around 20 million people, we routinely test adenovirus in nasopharyngeal swabs by RT-PCR assay or using serum IgM for in-hospital children with acute respiratory infection (ARI) since Jan 2011. In the present study, we retrospectively collected the children with respiratory adenoviral infection and identified the predictive factors of BO.

## Methods

### Patients

In the period between Jan 2011 and December 2014, the consecutive in-hospital ARI children with positive result for adenovirus in nasopharyngeal swabs by RT-PCR assay or serum IgM/IgG were enrolled into the present study. Diagnosis and classification of ARI followed standard WHO algorithm for ARI. Briefly, children with cough, difficult breathing, or both were diagnosed ARI and screened for fast breathing. Children were classified as Table 1 (WHO 1991; Hazir et al. 2011). Patients with one of following conditions were excluded from this study: patients presented congenital heart disease, congenital pulmonary dysplasia, immunodeficiency disease, malignances, severe organ dysfunction, had recently been pyrexial for 7 days before admission (temperature under the axillary is at or over 37.2 °C, had exhibited clinical evidence of active infection in other organs, had received corticosteroids within 1 week before admission for any reason and those with substantial missing data. The study was approved by the ethical committee of the Guangzhou Medical University, as well as the Guangzhou Women and Children’s Medical Center Hospital; written informed consent was obtained from the patients’ parents.

**Table 1:** World Health Organization classification of acute respiratory illness in children presenting with cough and/or difficult breathing

Classification	Criteria
No pneumonia (cough and cold)	Respiratory rate (breaths/minute)
	<50 (infants 2–11 months)
	<40 (children 12–59 months)
	No lower chest indrawing
Nonsevere pneumonia	Respiratory rate (breaths/minute)
	>50 (infants 2–11 months)
	<40 (children 12–59 months)
	No lower chest indrawing
Severe pneumonia	Lower chest indrawing with or without rapid breathing
Very severe disease	Unable to drink, convulsions, abnormally sleepy or difficult waking, stridor in calm child or clinically severe malnutrition

### Diagnosis of adenovirus infection

PCRs with DNA targets utilized 10 μl purified nucleic acid from Qiagen Quantitect Probe PCR kit (Qiagen, Crawley, UK). Thermal cycling conditions were as described previously, except for removal of the 50 °C hold for reverse transcription, and the enzyme activation hold at 95 °C was extended to fifteen minutes for the Qiagen Quantitect Probe PCR kit. All assays were performed in a Lightcycler 480 real-time PCR machine (Roche Diagnostics, Burgess Hill, UK). The Primers were forward “GCC ACG GTG GGG TTT CTA AAC TT”, reverse “GCC CCA GTG GTC TTA CAT GCA CAT C” and the sequence of the probe was “TGC ACC AGA CCC GGG CTC AGG TAC TCC GA” (Bezerra et al. 2011). Serum adenovirus IgM/IgG were tested using diagnostic ELISA kit (IMMUNOLAB GmbH, Kassel).

### Identification of BO

High resolution computerized tomography (HRCT) was utilized as the diagnostic technique for BO. It was conducted when children presented tachypnoea, increased anteroposterior chest diameter, crackles, wheezing or hypoxaemia for at least 30 days after the initial of acute respiratory infection. The chest HRCT was performed during quiet breathing and evaluated by 2 blinded radiologists. BO was defined on the presence of bronchiectasis and/or a mosaic pattern. Mosaic pattern was defined as segmental orlobular areas of hypoattenuation that are associated with narrowing of the caliber of the pulmonary vessels.

### Data collection and follow up

Data as follows were included: gender, age, history of breast-feeding, premature birth, number of siblings, extention of infection (no pneumonia, pneumonia), and blood tests before treatment such as blood neutrophil count, blood lymphocyte count, alanine aminotransferase (ALT), aspartate aminotransferase (AST), creatinine kinase (CK), CK-MB, lactate dehydrogenase (LDH), and C-reactive protein (CRP), as well as parameters identified after admission including hypoxemia, mycoplasma co-infection, bacterial co-infection, sepsis, usage of γ-globulin intravenously, use of antibiotics, use of glucocorticoids, mechanical ventilation, length of fever and length of hospital stay.

We conducted a routine follow-up from admission to 1 month after delivery and recorded the following in detail: symptoms, concomitant medications and adverse reactions. All the children were followed up regularly, either at the clinic or by telephone.

### Statistical analysis

The Kolmogorov–Smirnov test was used to evaluate the normality of distribution. Data were reported as median and range when distribution was not normal. Statistical differences in clinical characteristics between the 2 groups were compared using the *t* test, the Mann–Whitney test, and Fisher’s exact test. Group comparison tests were performed using the Wilcoxon rank-sum test. Multivariate analysis using a Logistic proportional hazards model was used to test for independent significance by backward elimination of insignificant baseline characteristics and explanatory variables. For all tests, a *p* value < 0.05 was considered statistically significant, and all *p* values quoted are 2-sided. Statistical analyses were performed using SPSS v. 20.0 (SPSS Inc., Chicago, IL, USA).

## Results

### Patient characteristics of children with or without BO following respiratory adenoviral infection

A total of 544 children (398 boys and 146 girls) met the eligibility criteria and were available for analysis. Among the 544 children, 456 children were diagnosed through PCR and 56 were by serum IgM with the rest 32 diagnosed by both methods. In the serology only group, seroconversion were confirmed in 47/56 (83.9 %) patients. The children had a median age of 23 months (range 1–144 months). 14 (2.57 %) patients developed BO who were all diagnosed pneumonia at admission. Compared with children without BO, BO children presented higher LDH (523.5 vs. 348 IU/ml, *p* = 0.033), lower blood lymphocyte count (2.23 × 10^9^/L vs. 3.24 × 10^9^/L, *p* = 0.025) and higher incidence of hypoxemia (78.6 vs. 20.8 %, *p* = 0.000). They presented relatively persistent fever (15.5 vs. 7 days, *p* = 0.000) and needed longer treatment in hospital (19.5 vs. 7 days, *p* = 0.000). Concerning treatment, they were given more intravenous γ-globulin (85.7 vs. 36.8 %, *p* = 0.000), glucocorticoids (78.6 vs. 24.3 %, *p* = 0.000) and mechanical ventilation (35.7 vs. 5.5 %, *p* = 0.001) (Table 2).

**Table 2:** Characteristics of children with or without bronchiolitis obliterans after respiratory adenoviral infection

Characteristics	Non-BO (n = 530)	BO (n = 14)	*p*
Gender			0.127
Male	385 (72.6 %)	13 (92.9 %)	
Female	145 (27.4 %)	1 (7.1 %)	
Age (months)	23.5 (1–144)	15.5 (6–72)	0.339
History of breast-feeding	305 (57.5 %)	9 (64.3 %)	0.818
Premature birth	34(6.4 %)	1 (7.1 %)	0.610
Number of siblings	0 (0–3)	1 (0–2)	0.096
Diagnosis			0.014
No pneumonia	150 (28.3 %)	0 (0.0 %)	
Pneumonia	380 (71.7 %)	14 (100.0 %)	
Hypoxemia	110 (20.8 %)	11 (78.6 %)	0.000
CRP	14.92 (0.00–264.74)	26.25 (0.51–116.20)	0.155
Blood neutrophil count (×10^9^/l)	5.49 (0.54–32.8)	4.46 (2.19–14.44)	0.767
Blood lymphocyte count (×10^9^/l)	3.24 (0.36–35.29)	2.23 (0.83–6.77)	0.025
ALT (IU/ml)	18 (3–372)	16 (7–112)	0.405
AST (IU/ml)	41 (15–1400)	40.5 (29–391)	0.495
CK (IU/ml)	88.5 (11–5413)	91 (17–1038)	0.941
CK–MB (IU/ml)	25 (1–436)	24 (13–169)	0.959
LDH (IU/ml)	348 (14–10983)	523.5 (254–1945)	0.033
Mycoplasma co-infection	143 (27.0 %)	4 (28.6)	0.999
Bacterial co-infection	46 (8.7 %)	0 (0.0 %)	0.621
Sepsis	9 (1.7 %)	0 (0.0 %)	0.999
Use of γ-globulin intravenously	195 (36.8 %)	12 (85.7 %)	0.000
Use of antibiotics	509 (96.0 %)	14 (100.0 %)	0.999
Use of glucocorticoids	129 (24.3 %)	11 (78.6 %)	0.000
Mechanical ventilation	29 (5.5 %)	5 (35.7 %)	0.001
Length of fever (days)	7 (0–62)	15.5 (6–30)	0.000
Length of hospital stay (days)	7 (2–74)	19.5 (8–55)	0.000

*BO* bronchiolitis obliterans, *ALT* alanine aminotransferase, *AST* aspartate aminotransferase, *CK* creatinine kinase, *LDH* lactate dehydrogenase, *CRP* C-reactive protein

### Predictors of BO following respiratory adenoviral infection in children

**Fig. 1:**
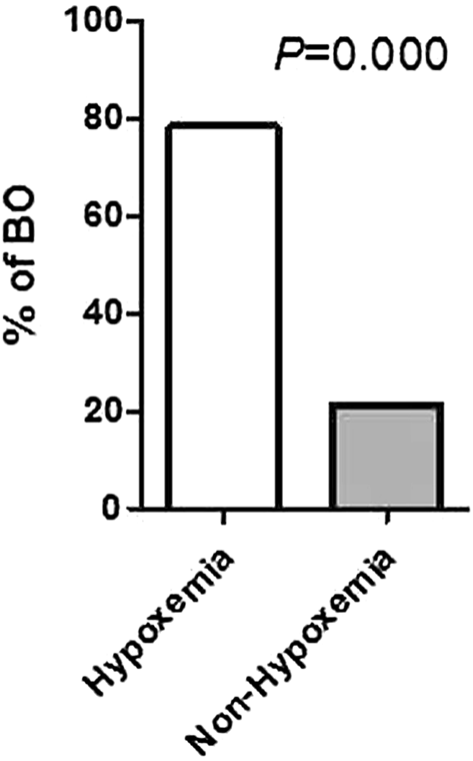
Incidence of BO in adenovirus infected children with or without hypoxemia. *BO* bronchiolitis obliterans

Univariate analysis by Logistic regression revealed that length of hospital stay (*p* = 0.000, HR 1.086, 95 % CI 1.046–1.127), length of fever (*p* = 0.003, HR 1.062, 95 % CI 1.020–1.106), use of glucocorticoids (*p* = 0.000, HR 11.398, 95 % CI 3.131–41.486), mechanical ventilation (*p* = 0.000, HR 9.598, 95 % CI 3.022–30.480), hypoxemia (*p* = 0.000, HR 14.000, 95 % CI 3.839–51.050) and use of intravenous γ-globulin (*p* = 0.002, HR 10.308, 95 % CI 2.283–46.535) were indicated as prognostic factors. Then, multiple analysis by Logistic regression using above parameters determined that hypoxemia was the only independent predictor for BO (*p* = 0.030, HR 5.046, 95 % CI 1.170–21.765) (Table 3). Then, patients with or without hypoxemia were compared concerning the incidence of BO, which indicated that patients with hypoxemia kept significantly higher incidence of BO than those without hypoxemia (Fig. 1).

**Table 3:** Logistic analysis of predictive parameters

Factors	Univariate analysis		Multivariate analysis	
	*p*	HR (95 % CI)	*p*	HR (95 % CI)
Length of hospital stay	0.000	1.086 (1.046–1.127)	0.056	1.044 (0.999–1.090)
Length of fever	0.003	1.062 (1.020–1.106)	0.997	1.000 (0.942–1.062)
Use of glucocorticoids	0.000	11.398 (3.131–41.486)	0.056	4.036 (0.964–16.907)
Mechanical ventilation	0.000	9.598 (3.022–30.480)	0.612	1.438 (0.354–5.849)
Hypoxemia	0.000	14.000 (3.839–51.050)	0.030	5.046 (1.170–21.765)
Use of γ-globulin intravenously	0.002	10.308 (2.283–46.535)	0.474	1.935 (0.317–11.806)

*95* *% CI* 95 % confidence interval

## Discussion

BO is a severe chronic sequelain children following respiratory adenoviral infection (Champs et al. 2011; Murtagh et al. 2009). Due to its low incidence, the predictors of BO were still unclear. Respiratory viral infection was confirmed to be a distinct risk for BO (Khalifah et al. 2004). Among the entire suspected virus, adenovirus was confirmed to be the major causes (Murtagh et al. 2009). Previous studies found that mechanical ventilation, >30 days of hospitalization, multifocal pneumonia and hypercapnia were the predictors of BO in children with acute bronchitis (Murtagh et al. 2009; Colom and Teper 2009; Yalcin et al. 2003; Khalifah et al. 2004). Some studies failed to identify the predictors due to limited sample sizes (Yalcin et al. 2003). Above all, few prognostic factors achieved worldwide acknowledgment, which might due to limited sample sizes and the heterogenicity of this disease.

In the present study, we found that hypoxemia was the independent predictor of BO in adenovirus infected children, which was a novel finding. It usually preceded the previous reported risk factors such as hypercapnia and mechanical ventilation (Jouett et al. 2015; Wilson and Matthay 2014), which made it more sensitive than other predictors. The causes and mechanism of development of BO was still unclear (Kim et al. 2006; Gedik et al. 2015; Costa et al. 2005; Mallol et al. 2011). In order to achieve reliable results, prognostic analysis of BO shall be based on simple disease background. Since adenoviral infection was identified as the major cause of BO in children (Murtagh et al. 2009), it is optimal to investigate the predictors for BO following respiratory adenoviral infection in children. Due to the low incidence of BO, multiple center based studies shall be the optimal choice for better understanding of this sequela.

In the present study, pulmonary function measurements were not conducted due the ages of BO children. Most of them are younger than 5 years, which made the pulmonary function measurements unreliable due to their incompatibility. Meanwhile, pathological diagnosis was not achieved due to the risk of biopsy in symptomatic young children and reluctance of their parents. However, the diagnosis of BO in most of the studies was not based on pathological diagnosis (Li et al. 2014; Gedik et al. 2015; Giubergia et al. 2015). And diagnosis based on CT and clinical features was acceptable to most of the researchers. We did not test co-infection of other virus and specific types of adenovirus, due to the low incidence of BO and sub-analysis needed multi-center based studies with larger sample sizes.

## Conclusions

In summary, the present study identified hypoxemia as the independent predictive factor of BO in adenoviral infected children, which was a novel and sensitive predictor.

## References

[CR1] Bezerra PG, Britto MC, Correia JB, Duarte Mdo C, Fonceca AM, Rose K (2011). Viral and atypical bacterial detection in acute respiratory infection in children under five years. PLoS ONE.

[CR2] Bosa VL, Mello ED, Mocelin HT, Benedetti FJ, Fischer GB (2008). Assessment of nutritional status in children and adolescents with post-infectious bronchiolitis obliterans. J Pediatr (Rio J).

[CR3] Champs NS, Lasmar LM, Camargos PA, Marguet C, Fischer GB, Mocelin HT (2011). Post-infectious bronchiolitis obliterans in children. J Pediatr (Rio J).

[CR4] Colom AJ, Teper AM (2009). Clinical prediction rule to diagnose post-infectious bronchiolitis obliterans in children. Pediatr Pulmonol.

[CR5] Costa ML, Stein RT, Bauer ME, Machado DC, Jones MH, Bertotto C, Pitrez PM (2005). Levels of Th1 and Th2 cytokines in children with post-infectious bronchiolitis obliterans. Ann Trop Paediatr.

[CR6] El-Gamel A, Sim E, Hasleton P, Hutchinson J, Yonan N, Egan J, Campbell C, Rahman A, Sheldon S, Deiraniya A, Hutchinson IV (1999). Transforming growth factor beta (TGF-beta) and obliterative bronchiolitis following pulmonary transplantation. J Heart Lung Transplant.

[CR7] Gedik AH, Cakir E, Gokdemir Y, Uyan ZS, Kocyigit A, Torun E, Karadag B, Ersu R, Karakoc F (2015) Cathelicidin (LL-37) and human β2-defensin levels of children with post-infectious bronchiolitis obliterans. Clin Respir J. doi:10.1111/crj.12331 10.1111/crj.12331 26073571

[CR8] Giubergia V, Salim M, Fraga J, Castiglioni N, Sen L, Castanos C, Mangano A (2015). Post-infectious bronchiolitis obliterans and mannose-binding lectin insufficiency in Argentinean children. Respirology.

[CR9] Hadjiliadis D, Davis RD, Palmer SM (2002). Is transplant operation important in determining posttransplant risk of bronchiolitis obliterans syndrome in lung transplant recipients?. Chest.

[CR10] Hazir T, Nisar YB, Abbasi S, Ashraf YP, Khurshid J, Tariq P (2011). Comparison of oral amoxicillin with placebo for the treatment of world health organization-defined nonsevere pneumonia in children aged 2–59 months: a multicenter, double-blind, randomized, placebo-controlled trial in pakistan. Clin Infect Dis.

[CR11] Heng D, Sharples LD, McNeil K, Stewart S, Wreghitt T, Wallwork J (1998). Bronchiolitis obliterans syndrome: incidence, natural history, prognosis, and risk factors. J Heart Lung Transplant.

[CR12] Husain AN, Siddiqui MT, Holmes EW, Chandrasekhar AJ, McCabe M, Radvany R, Garrity ER (1999). Analysis of risk factors for the development of bronchiolitis obliterans syndrome. Am J Respir Crit Care Med.

[CR13] Jouett NP, Watenpaugh DE, Dunlap ME, Smith ML (2015). Interactive effects of hypoxia, hypercapnia and lung volume on sympathetic nerve activity in humans. Exp Physiol.

[CR14] Khalifah AP, Hachem RR, Chakinala MM, Schechtman KB, Patterson GA, Schuster DP, Mohanakumar T, Trulock EP, Walter MJ (2004). Respiratory viral infections are a distinct risk for bronchiolitis obliterans syndrome and death. Am J Respir Crit Care Med.

[CR15] Kim DK, Yoo Y, Yu J, Choi SH, Koh YY (2006). Bronchial responsiveness to methacholine and adenosine 5’-monophosphate (AMP) in young children with post-infectious bronchiolitis obliterans. Acta Paediatr.

[CR16] Li YN, Liu L, Qiao HM, Cheng H, Cheng HJ (2014). Post-infectious bronchiolitis obliterans in children: a review of 42 cases. BMC Pediatr.

[CR17] Mallol J, Aguirre V, Espinosa V (2011). Increased oxidative stress in children with post infectious Bronchiolitis Obliterans. Allergol Immunopathol (Madr).

[CR18] Mosquera RA, Hashmi SS, Pacheco SE, Reverdin A, Chevallier J, Colasurdo GN (2014). Dysanaptic growth of lung and airway in children with post-infectious bronchiolitis obliterans. Clin Respir J.

[CR19] Murtagh P, Giubergia V, Viale D, Bauer G, Pena HG (2009). Lower respiratory infections by adenovirus in children. Clinical features and risk factors for bronchiolitis obliterans and mortality. Pediatr Pulmonol.

[CR20] WH Organization (1991) Technical basis for the WHO recommendations on the management of pneumonia in children at first level health facilities. Geneva: World Health Organization. WHO/ARI/91.20

[CR21] Palmer SM, Davis RD, Hadjiliadis D, Hertz MI, Howell DN, Ward FE, Savik K, Reinsmoen NL (2002). Development of an antibody specific to major histocompatibility antigens detectable by flow cytometry after lung transplant is associated with bronchiolitis obliterans syndrome. Transplantation.

[CR22] Wilson JG, Matthay MA (2014). Mechanical ventilation in acute hypoxemic respiratory failure: a review of new strategies for the practicing hospitalist. J Hosp Med.

[CR23] Xie BQ, Wang W, Zhang WQ, Guo XH, Yang MF, Wang L, He ZX, Tian YQ (2014). Ventilation/perfusion scintigraphy in children with post-infectious bronchiolitis obliterans: a pilot study. PLoS ONE.

[CR24] Yalcin E, Dogru D, Haliloglu M, Ozcelik U, Kiper N, Gocmen A (2003). Postinfectious bronchiolitis obliterans in children: clinical and radiological profile and prognostic factors. Respiration.

